# Prevalence and Genotyping of *Trichomonas gallinae* in Pigeons in the Mashhad Area, Khorasan Razavi Province, Iran

**DOI:** 10.1002/vms3.71004

**Published:** 2026-05-21

**Authors:** Zahra Hajiannejad, Hossein Nourani, Gholamreza Razmi

**Affiliations:** ^1^ Department of Pathobiology, Faculty of Veterinary Medicine Ferdowsi University of Mashhad Mashhad Iran

**Keywords:** epidemiology, phylogeny, pigeon, trichomonosis

## Abstract

**Background:**

Trichomonosis is one of the most common gastrointestinal infections found in pigeons worldwide. Although many studies in Iran have examined the prevalence of *Trichomonas gallinae* infections in pigeons and various bird species, there have been relatively few studies investigating the genotypes of this protozoan.

**Objectives:**

The purpose of this study was to examine *T. gallinae* infection in pigeons in the Mashhad area, Iran, using parasitological, pathological and genetic methods.

**Methods:**

Between April and July 2022, a total of one hundred oral swabs were gathered for microscopic, culture and molecular analysis. Additionally, deceased pigeons were transported to the laboratory for necropsy and histopathological examinations. Sequence analysis was performed on five polymerase chain reaction (PCR) products from *T. gallinae* based on the amplification of the *ITS1/5.8S/ITS2* gene to identify their genotype.

**Results:**

Out of 100 collected samples, *T. gallinae* infection was detected in 50 samples using microscopic examinations, culture and PCR methods. Among the infected pigeons, 49 exhibited no clinical symptoms, whereas only one pigeon presented with severe clinical symptoms and ultimately succumbed to the illness. The macroscopic assessment revealed the presence of yellow caseous necrotic material in the oral cavity and pharynx. Histopathological analysis demonstrated infiltration of mononuclear cells and hyperaemia within the mucosal tissues of the oral cavity and oesophagus and crop, additionally, necrotic lesions and hyperaemia were identified in the liver. The sequencing and phylogenetic examination of the internal transcribed spacer (*ITS*) region from five isolates in this study reveal a distinct lineage of *T. gallinae* present in pigeons located in the Mashhad area.

**Conclusions:**

The study results indicated a high prevalence of *T. gallinae* infection in pigeons, and for the first time, a distinct lineage of *T. gallinae* from pigeons in Iran was documented.

## Graphical abstract

Prevalence and genotyping were conducted to determine *Trichomonas gallinae* infections among domestic pigeons in the Mashhad region of Iran. A total of 100 collected samples, *T. gallinae* infection was identified in 50 samples through microscopic parasitological, pathological and PCR methods. Phylogenetic analysis showed a distinct lineage of *T. gallinae*.

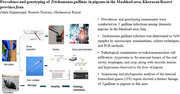

**Abbreviations**
*ITS*
: internal transcribed spacer
PCR: polymerase chain reaction

## 1 Introduction

*Trichomonas gallinae* causes trichomonosis in domestic and wild pigeons, doves and raptors and rarely in waterfowl. The protozoan has an elongated‐oval or pear‐shaped body with four anterior flagella that originate from the kinetosome. The posterior flagellum does not reach the end of the body, and there is no free flagellum (Taylor et al. 2013; Soulsby 1982; Calnek et al. 1997). The disease is transmitted to birds through various routes, including crop milk, infected water and feed; aggregation at bird feeders or contaminated birdbaths; and the consumption of infected prey (Taylor et al. 2013; Calnek et al. 1997).

The adult pigeons may remain subclinical while carrying the infection for extended periods, often up to a year or more, thereby consistently exposing their young to the disease (Taylor et al. 2013; Calnek et al. 1997). *T. gallinae* resides in the upper digestive system, particularly the oesophagus and crop, although it can also invade the lungs, liver, internal body lining, air sacs, pancreas, bones and skull sinuses (Taylor et al. 2013; Calnek et al. 1997). Most strains of *T. gallinae* are non‐pathogenic with mild signs. Pathogenic strains of this parasite cause abundant yellow necrotic lesions on the choana, tongue or pharyngeal mucosa; sometimes they spread systemically to other organs (Taylor et al. 2013; Calnek et al. 1997). The clinical signs of the disease include the loss of transparency of feathers, loss of appetite, discharge of very foul‐smelling yellowish‐green discharge, diarrhoea, emaciation, severe weakness and death (Taylor et al. 2013; Calnek et al. 1997). The pathological studies were demonstrated the presence of extensive caseous necrosis along with widespread infiltration of mononuclear cells and congestion within the lamina propria of the larynx, oesophagus and lungs of infected pigeons. Additionally, they identified significant aggregation of mononuclear cells and heterophils, along with necrosis in the parenchyma, as well as congestion and dilation in the hepatic sinusoids, and the formation of granulomas consisting of mononuclear cells and heterophils (Begum et al. 2008; Borji et al. 2011; Fadhil et al. 2020).

The classification of *T. gallinae* gene types based on the *ITS1/5.8S/ITS2* regions has been documented in various ways within the scientific community, predominantly through three separate nomenclature frameworks. Gerhold et al. (2008) proposed the first framework, which categorized genotypes as A, B, C, D and L. Martínez‐Herrero et al. (2014) presented the second framework, using identifiers such as internal transcribed spacer *(ITS)‐OBT‐Tg‐1* and *ITS‐OBT‐Tg‐2*. The third framework, defined by Grabensteiner et al. (2010), referred to the categories as *ITS‐I* and *ITS‐II*. In a later investigation, Marx et al. (2017) confirmed that lineage C/V/N as “genotype A” and lineage A/B as ‘genotype B’, while also introducing additional lineages O, P and Q.

On the basis of the phylogenetic analysis of the ITS gene region of *T. gallinae*, 2 genotypes in Iran (Arabkhazaeli et al. 2019; Ayati et al. 2023), 1 genotype in Iraq (Al‐Sadi and Hamodi 2011), 15 genotypes in Saudi Arabia (Albeshr and Alrefaei 2020), 3 genotypes in China (Jing et al. 2024; Cai et al. 2022), 1 genotypes in Japan (Chou et al. 2022), 2 genotypes in Egypt (El‐Khatam et al. 2016), 12 genotypes in USA (Gerhold et al. 2008) and 6 genotypes in European birds (Grabensteiner et al. 2010) have been reported. A comprehensive analysis indicated that the host species plays a crucial role in determining the variations observed in *T. gallinae* infection rates (Liu et al. 2024).

In Iran, the majority of research has focused on the prevalence of *T. gallinae* among pigeons across various provinces (Ahmadabad and Nasrabadi 2024), whereas there has been limited investigation into the identification of *Trichomonas* genotypes in pigeons and other birds (Arabkhazaeli et al. 2019; Ayati et al. 2023).

The objective of this study was to assess the prevalence of infection and to investigate the clinical signs and pathological changes observed in pigeons in the Mashhad area, Khorasan Razavi province, Iran. Furthermore, the research sought to identify the genotype of the strain responsible for this infection.

## 2 Materials and Methods

### 2.1 Sampling

In this study, one hundred domestic pigeons were sampled from pigeon houses in some parts of the Mashhad area. Each pigeon's oral cavity was inspected for any lesions, and if found, they were noted along with other clinical symptoms. Two oral swabs were prepared for each pigeon. A damp swab was inserted into the pigeon's mouth and gently moved around for a few seconds before placing it in a sterile tube with 1 mL of Ringer's solution. The specimens were conveyed to the parasitology laboratory under controlled cool conditions. Furthermore, deceased pigeons exhibiting oral injuries were transported to the laboratory for necropsy and histopathological examination.

### 2.2 Microscopic Examination

In the laboratory, wet mount and stained smears with the Giemsa method were prepared in the laboratory. Wet smears were examined with a light microscope at ×100 and ×400 magnifications. Moreover, smears were stained and studied with an oil lens for *Trichomonas* detection.

### 2.3 Culture in Dorset Medium

The Dorset medium is biphasic, consisting coagulated eggs slants as solid phase and 5 mL serum ringer, antibiotics and with rice starch as liquid. The oral swab of each infected pigeon was inoculated in liquid phase. The culture was kept in an incubator at 37° for a week and examined for growth on Days 3–7 post culture (Badparva et al. 2010). Following this period, the serum media was meticulously transferred to a tube containing physiological serum with a volume of 3 mL, utilizing a Pasteur pipette. The sample was then centrifuged at 2000 rpm for duration of 5 min. The supernatant was discarded and repeated washing two times. The washed sediment at the bottom of the tube was retrieved using a sampler and placed into a microtube, which was then stored in the laboratory freezer until the DNA extraction procedure commenced.

### 2.4 Necropsy and Histopathology

In this study, the pigeon carcass was dissected and tissue samples with lesions found in the oral cavity, oesophagus, crop, proventriculus and liver were collected and placed in a 10% neutral buffered formalin container. The fixed tissues were processed through a tissue processor and embedded in paraffin wax. Following the preparation of the blocks, sections were cut serially to a thickness of 5 µm using a microtome. The sections were then deparaffinized and stained with haematoxylin and eosin (H&E) for subsequent histopathological analysis under light microscopy.

### 2.5 DNA Extraction and Polymerase Chain Reaction (PCR) Test

The collected samples were extracted using an animal tissue DNA extraction kit (Dena zist Company, Mashhad) according the kit's instructions. A PCR assay was carried out to detect the *T. gallinae* gene as previously described based on the Felleisen method with amplification of *ITS1/5.8S/ITS2* fragment (Felleisen 1997). The simple PCR reaction was performed in a 25 µL mixture containing 2 µL of total DNA, 12.5 µL of commercial premix master mix (Parstous Mashhad), 1 µL of each primer and 8.5 µL of nuclease‐free water in a thermocycler. The cycling condition was as follows: an initial denaturation step at 94°C for 5 min followed by 35 cycles of 94°C for 1 min, 67°C for 30 s, 74°C for 1 min and a final extension step at 74°C for 5 min. The oligonucleotide primers were forward primer (5′‐TGCTTCAGTTCAGCGG GTCTTCC‐3′) and reverse primer: (5′‐CGGTAGGTGAACCTGCCGTTGG‐3′). The distilled water was added instead of the DNA sample in the negative control microtube. The positive control sample was prepared from *T. gallinae*, which was previously grown in Dorsett medium. The resulting products were subjected to electrophoresis on a 2% agarose gel in TBE ×1 buffer and visualized using ultraviolet light. The size of the DNA fragments was compared with a standard molecular weight (100 bp DNA ladder). The positive control sample had a band of 369 bp.

### 2.6 Gene Sequencing and Phylogeny

Among all PCR‐positive samples, four samples of pigeons without symptoms and one sample of pigeons with symptoms with high DNA concentration were chosen. Following packing, the DNA samples with use aforementioned primers were submitted for sequencing to a company in Iran (Topaz‐ Genekaush Co, Karj, Iran). The nucleotide sequences were assembled and edited with the Mega software Version 11. The edited nucleotide sequences of isolates were aligned with previously sequences of *T. gallinae* in GenBank (NCBI) using the Claustral W method (Mega software Version 11). The phylogenetic analysis was carried out with the neighbour‐joining process and bootstraps of 1000 replication.

## 3 Results

**FIGURE 1:**
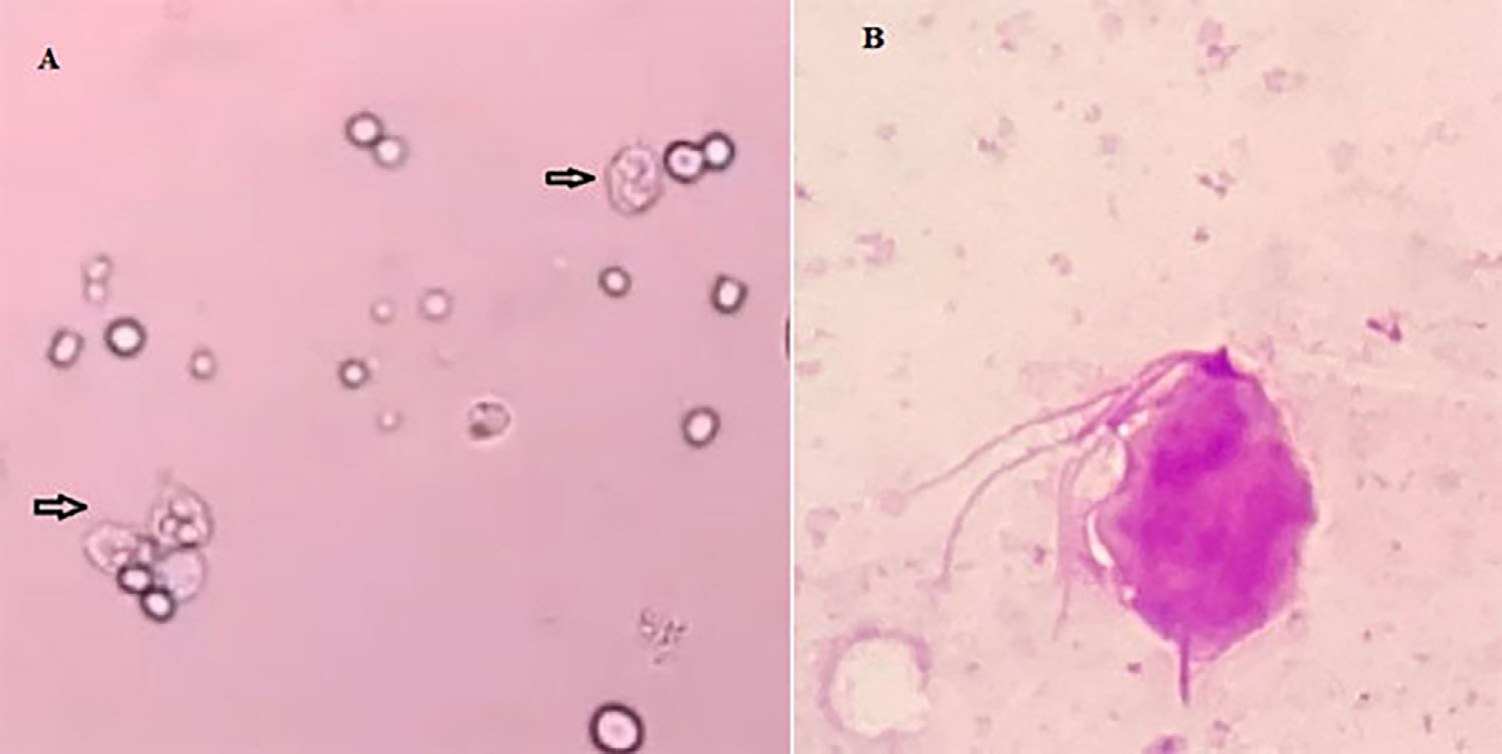
*Trichomonas gallinae*. (A): unstained and (B) stained *T.gallinae*.

**FIGURE 2:**
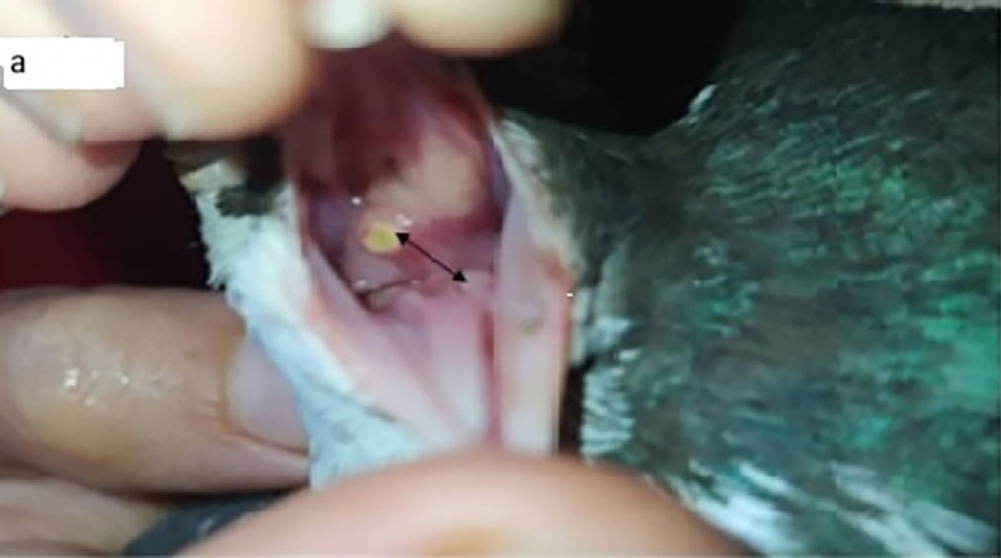
The presence of cheesy lesions and pus in the mouth of a sick pigeon (double‐sided arrow).

**FIGURE 3:**
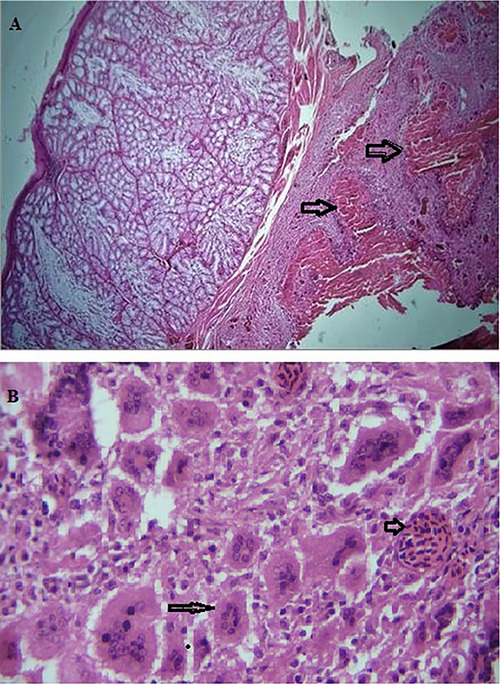
(A) Caseous necrosis observed in the crop of a pigeon, accompanied by inflammatory cells (arrow) (40× magnification). (B) The presence of inflammatory cells along with giant cells (arrow) and hyperaemia (arrow) in the crop (40× magnifications).

**FIGURE 4:**
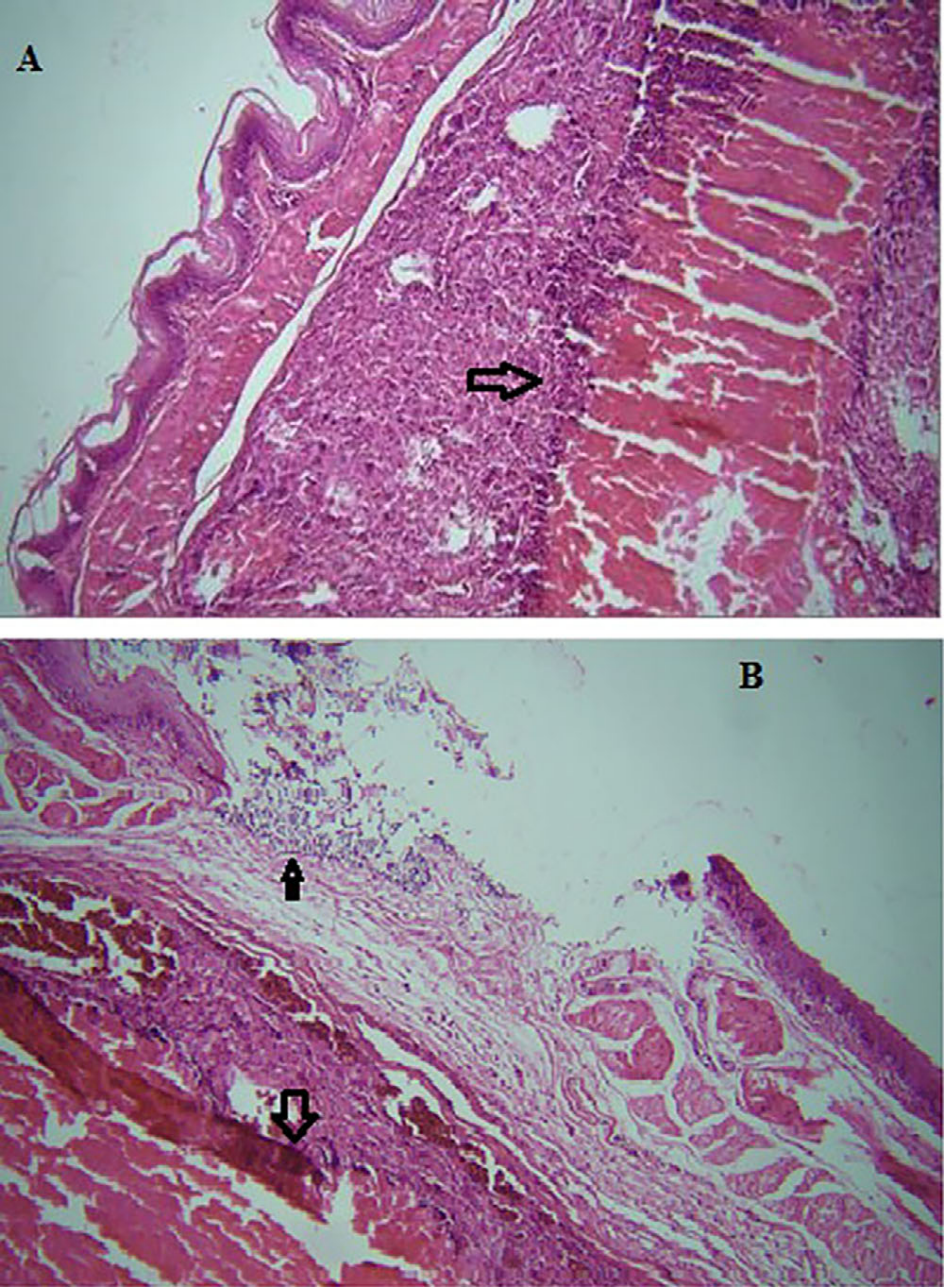
(A) Caseous necrosis (arrow) and inflammation under the lining of the oesophagus (100× magnifications). (B) An ulcer is present, accompanied by hyperaemia, bleeding and the clustering of inflammatory cells around necrotic tissue in the mucosa (indicated by an arrow) and muscular layers (also indicated by an arrow) of the oesophagus (100× magnification).

**FIGURE 5:**
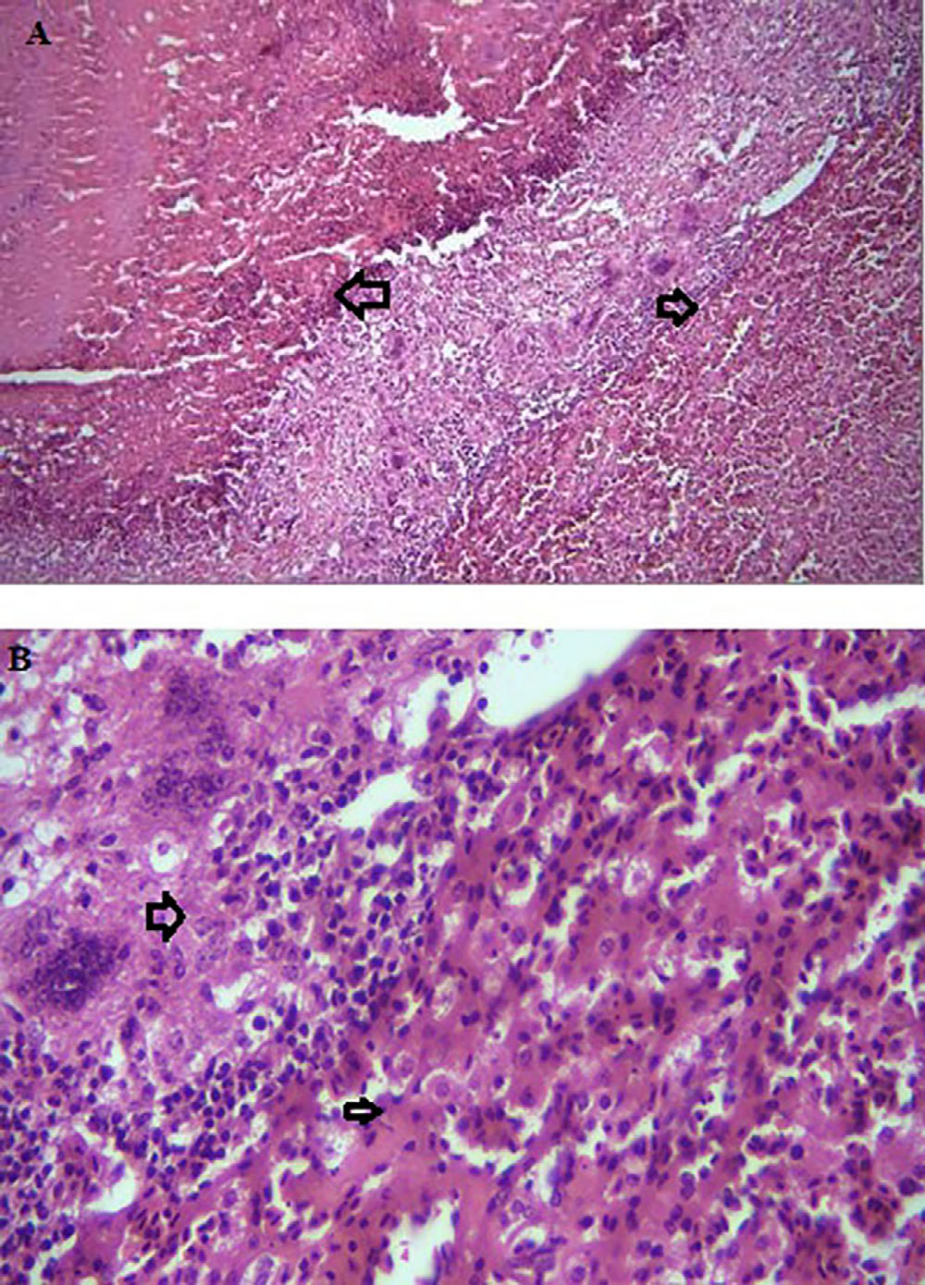
(A) Necrosis (arrow) and severe hyperaemia (arrow) in the liver (100× magnification). (B) Necrosis, accumulation of inflammatory cells (arrow) and severe hyperaemia (arrow) in the liver (400× magnification).

In the present study, *T. gallinae* were microscopically detected in wet mount 50% (Figure 1). Out of 50 infected pigeons, 49 pigeons had no clear clinical symptoms, and only one pigeon had clear clinical symptoms such as lethargy, weakness and emaciation, and cheesy purulent lesions were seen in mouths and throats (Figure 2). The pigeon was succumbed following a short duration of sampling. The histopathological examination revealed caseous necrosis with a localized accumulation of lymphocytes and macrophages beneath the mucus accompanied by bleeding in the crop (Figure 3A,B). Furthermore, the examination noted the presence of heterophil infiltration beneath mucosa, along with hyperaemia and the infiltration of mononuclear cells and within the muscle layers of the oesophageal tissue (Figure 4A,B). Additionally, necrotic lesions and hyperaemia were identified in the liver (Figure 5A,B).

**FIGURE 6:**
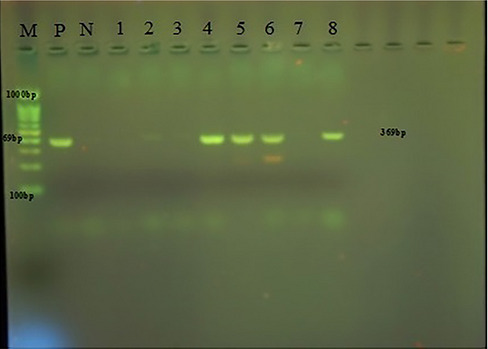
Detection of *Trichomonas gallinae* by PCR method with specific primers: (M): 100 bp marker, (P): positive control with 369 bp, (N): negative control, 2–6 and 8: positive samples, s 1 and 7: negative samples.

All samples that tested positive under the microscope were effectively cultured in Dorsett medium, and PCR analysis verified the presence of *Trichomonas* infection in those cultures. In the study, PCR detected positive results in 46 out of 50 cultured samples. One sample was related to sick pigeons with clinical symptoms and the other was related to pigeons without clinical signs (Figure 6). The sequences have been submitted to GenBank under access number PQ 218597.1 for Isolate 5, which pertains to a pigeon exhibiting clinical signs, and under access numbers PQ 226391.1, PQ 219391.1, PQ 219679.1 and PQ 219682.1 for isolates 1 through 4, corresponding to pigeons that do not display clinical signs.

**FIGURE 7:**
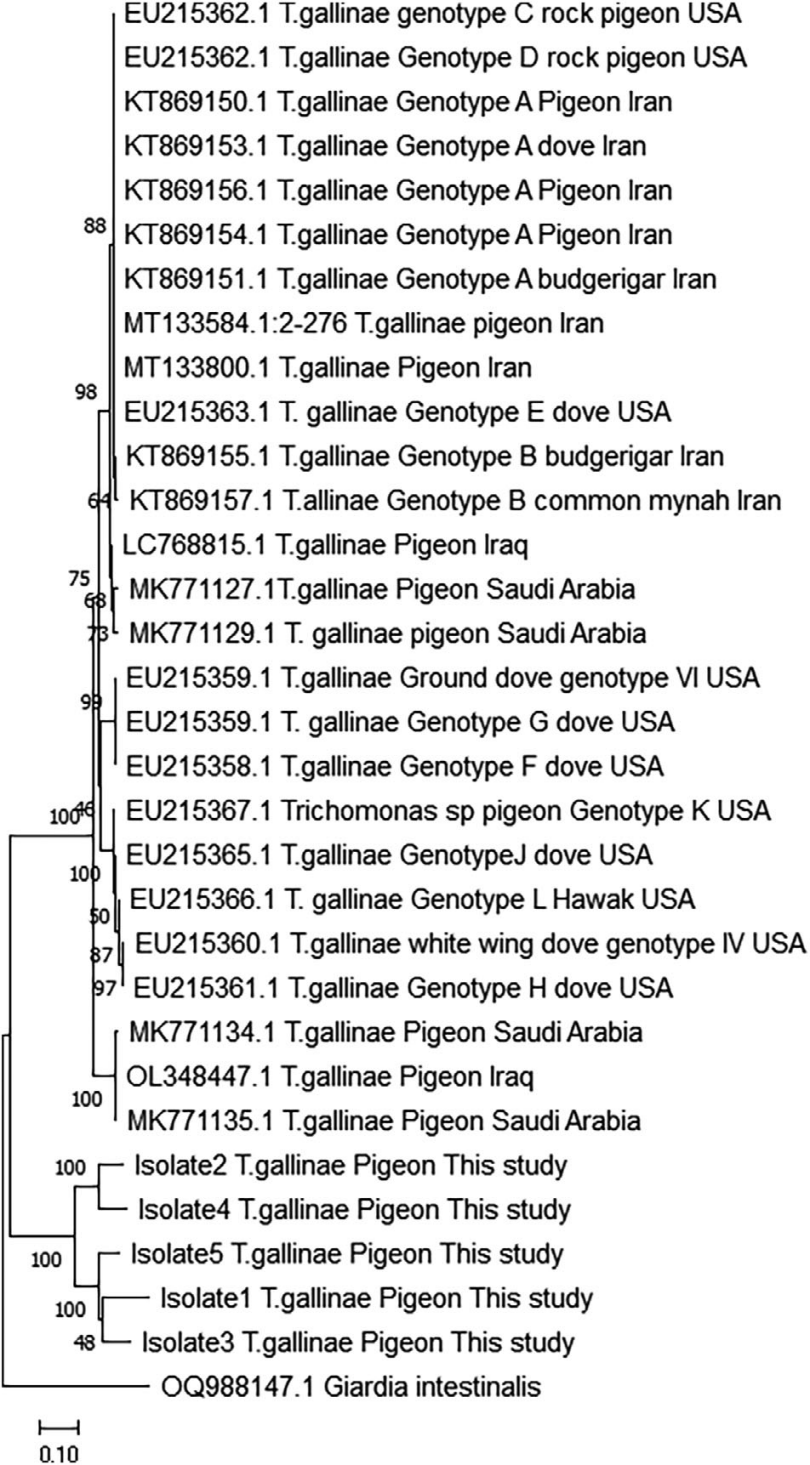
The phylogenetic tree of the isolates identified in this study with the sequenced samples of *Trichomonas gallinae* in Iran and other countries based on ITS1/5.8S/ITS2 gene with the neighbour‐joining method with a bootstrap of 1000 replication.

The comparison of the nucleotide sequences showed that the *T. gallinae* nucleotide sequences have similarities ranging from 83% to 97% in our samples. Additionally, the analysis of the phylogenetic tree supported the differences between the genotypes of the isolates in this study and those of *T. gallinae* genotypes reported in Iran and other countries (Figure 7).

## 4 Discussion

In this study, the frequency of *T. gallinae* infection was determined at 50% in domestic pigeons using a microscopic method. Direct microscopic examination of wet mounts or stained smears in pigeons from different provinces of Iran has reported a high frequency of *T. gallinae* infection. Briefly, the prevalence of *T. gallinae* was reported at 78.94% in Ahvaz city, Khuzestan Province (Miyahi et al. 2007), 37.32% in the Mashhad area, Khorasan Razavi Province (Borji et al. 2011), 57% in Isfahan Province (Nematollahi et al. 2012), 72.7% in Zabul city, Sistan and Baluchistan Province (Samiee et al. 2016),70.90% in Shahrekord Province (Pirali‐Kheirabadi et al. 2008) and 53% in Western Azerbaijan Province (Adinehbeigi et al. 2018). The high prevalence of subclinical signs infection in pigeons supports this bird acts as a reservoir host.

The variation in reported prevalence can be attributed to several factors including age, weather, the population of the flock, the form of breeding, inadequate housing sanitation, the provision of contaminated food and water and the presence of carriers in the flock.

Different prevalences of *T. gallinae* infection have been reported in neighbouring and other countries, including Iraq (Al‐Sadi and Hamodi 2011; Fadhil and Faraj 2019; Hammadi et al. 2023), Saudi Arabia (Albeshr and Alrefaei 2020); Pakistan (Saleem et al. 2008), Bangladesh (Begum et al. 2008), China (Jing et al. 2024; Cai et al. 2022), Egypt (El‐Khatam et al. 2016; Mohamed et al. 2023) and Europe (Marx et al. 2017) in pigeons. Out of 50 infected pigeons, 49 had no clinical signs, and only one pigeon was sick with clinical signs. Out of 50 infected pigeons, 49 had no clinical signs, and only one pigeon was sick with clinical signs.

Avian trichomoniasis, caused by *T. gallinae*, is a disease of young pigeons (Calnek et al. 1997). The low incidence of clinically symptomatic pigeons in this study may be due to the fact that the sample was collected from adult pigeons. In this study, the white to yellowish material covered the oral mucosa. Microscopically, these caseous masses revealed an infiltrate of mononuclear cells, neutrophils, several giant cells, hyperaemia and necrosis in the oral cavity, oesophagus, crop and liver. Comparable lesions have been documented in analogous studies on pigeons in Iran and other countries (Borji et al. 2011; Samiee et al. 2016; Begum et al. 2008; Fadhil et al. 2020). Many media cultures support the growth of *T. gallinae*, the results demonstrated that growth media for trichomonads detection is more sensitive than wet mount preparations (Bunbury et al. 2005; Raza et al. 2018). Among different media cultures, the components for the Dorsett medium can be made inexpensively within the culture medium field, and it provides sensitivity comparable to that of the diamond medium (Iqbal‐Qureshi et al. 2002). The isolation and cultivation of *T. gallinae* from all oral swabs collected from infected pigeons were effectively achieved in this research using a Dorsett medium.

Four culture‐positive samples were negative in the PCR analysis. Several studies have examined the sensitivity and specificity of culture and PCR techniques for the diagnosis of *Trichomonas vaginalis*. The findings indicated that both methods exhibit high sensitivity and specificity. Nonetheless, there were instances where *T. vaginalis* infection was identified in the culture medium, yet the PCR test returned a negative result. Conversely, there were also cases in which the PCR test produced a positive result while the culture test yielded a negative outcome (Radonjic et al. 2006; Crucitti et al. 2003). They explained that the negative PCR findings could potentially be attributed to the inability of the existing primers to detect the parasite DNA present or to the possibility of variations within *T. vaginalis*, and also the presence of inhibitors in some media (Radonjic et al. 2006; Crucitti et al. 2003).

On the basis of the phylogenetic analysis of the ITS gene region of *T. gallinae*, 2 genotypes in Iran (Arabkhazaeli et al. 2019; Ayati et al. 2023), 1 genotype in Iraq (Al‐Sadi and Hamodi 2011), 15 genotypes in Saudi Arabia (Albeshr and Alrefaei 2020), 3 genotypes in China (Jing et al. 2024; Cai et al. 2022), 1 genotypes in Japan (Chou et al. 2022), 2 genotypes in Egypt (El‐Khatam et al. 2016), 12 genotypes in USA (Gerhold et al. 2008) and 6 genotypes in European birds (Grabensteiner et al. 2010) have been reported. A comprehensive analysis indicated that the host species plays a crucial role in determining the variations observed in *T. gallinae* infection rates (Liu et al. 2024).

The evolutionary analysis of the *ITS1/5.8S/ITS2* gene sequences of five *T. gallinae* isolates in the Mashhad area formed a separate clade, which was different from genotypes A to L found in pigeons and other birds in Iran and other countries (Arabkhazaeli et al. 2019; Ayati et al. 2023; Gerhold et al. 2008; Chou et al. 2022; Liu et al. 2024). It seems that these isolates belong to a distinct lineage and need further investigation. In this study, there was no observed correlation between the virulence and genotype of isolates derived from both healthy and diseased infected pigeons. The findings from the studies on this topic are highly inconsistent. Some studies indicated a correlation between the genotype of *T. gallinae* and its virulence (Lawson et al. 2011; Ganas et al. 2014), whereas other studies did not identify such a correlation (Sansano‐Maestre et al. 2009; Rijks et al. 2019).

In this study, the frequency of *T. gallinae* infection was high in the pigeons in the Mashhad area, Iran. The obtained results showed that the isolates’ nucleotide sequences in the ITS1/5.8S/ITS2 segment had a similarity range of 83%–97%. Upon analysing the nucleotide sequence and constructing the phylogenetic tree, it was confirmed that there was a single genotype among the infected pigeons. Additionally, these isolates were found to be distinct from the sequences identified in Iran and other countries.

## Author Contributions

Conceptualization: Gholamreza Razmi. Methodology: Zahra Hajiannejad, Gholamreza Razmi and Hossein Nourani. Investigation: Zahra Hajiannejad, Gholamreza Razmi and Hossein Nourani. Writing – original draft preparation: Gholamreza Razmi. Writing – review and editing: Gholamreza Razmi and Hossein Nourani. Funding acquisition: Gholamreza Razmi. Supervision: Gholamreza Razmi.

## Funding

This study was supported by grant 3/56894 from the Vice President of Research and Technology of Veterinary Medicine, Ferdowsi University of Mashhad, Iran.

## Ethics Statement

All experiments involving pigeons were conducted in full compliance with the guidelines sanctioned by the Animal Ethics Committee of our faculty, reference number IR.UM.REC.1400.363.

## Conflicts of Interest

The authors declare no conflicts of interest.

## Data Availability

The datasets generated during and/or analysed during the current study are available from the corresponding author upon reasonable request.
